# Synthesis and molecular docking studies of some novel Schiff bases incorporating 6-butylquinolinedione moiety as potential topoisomerase IIβ inhibitors

**DOI:** 10.1098/rsos.172407

**Published:** 2018-06-20

**Authors:** Hany M. Hassanin, Rabah A. T. Serya, Wafaa R. Abd Elmoneam, Mai A. Mostafa

**Affiliations:** 1 Department of Chemistry, Faculty of Education, Ain Shams University Roxy Cairo 11711, Egypt; 2 Pharmaceutical Chemistry Department, Faculty of Pharmacy, Ain Shams University, Cairo 11566, Egypt

**Keywords:** molecular docking, Schiff bases, pyrane, quinolone, topoisomerase, anti-cancer

## Abstract

A series of novel pyranoquinolinone-based Schiff's bases were designed and synthesized. They were evaluated for topoisomerase IIβ (TOP2B) inhibitory activity, and cytotoxicity against breast cancer cell line (MCF-7) for the development of novel anticancer agents. A molecular docking study was employed to investigate their binding and functional properties as TOP2B inhibitors, using the Discovery Studio 2.5 software, where they showed very interesting ability to intercalate the DNA–topoisomerase complex. Compounds **2a**, **2c** and **2f** showed high docking score values (82.36% −29.98 kcal mol^−1^ for compound **2a**, 78.18% −26.98 kcal mol^−1^ for compound **2c** and 78.65, −28.11 kcal mol^−1^ for compound **2f**) and revealed the highest enzyme inhibition activity. The best hit compounds exhibited highly potent TOP2B inhibitors with submicromolar IC50 at 5 µM compared to the reference doxorubicin.

## 1. Introduction

Topoisomerases are crucial enzymes that control the higher-order structural state of DNA. By selective cleaving, the problems of DNA are resolved by temporarily cleaving both strands of a DNA duplex to form a cleavage complex through which another DNA segment can be transported [1]. Topoisomerases can be classified in two general categories, termed type I or type II, depending on whether one or both DNA strands of a single duplex are cleaved during a catalytic cycle, respectively [2]. A variety of small-molecule agents capable of inducing such effects are widely prescribed as anti-cancer drugs which in turn increase the population of topoisomerase II (TOP2) breaking complexes, which leads to TOP2-mediated chromosome DNA cleavage and ultimately death of cancer cells [3]. Type II topoisomerases operate by a complicated mechanism that involves the controlled association and dissociation of subunit dimerization elements [4]. In humans TOP2 is further expressed into two type IIA topoisomerase isoforms, termed topoisomerase II*α* (TOP2A) and topoisomerase IIβ (TOP2B) [5,6]. Type IIA topoisomerases share significant amino acid sequence similarity between species. TOP2A plays a crucial significant role in chromosome segregation and DNA replication [7,8], whereas TOP2B appears to be involved mainly in transcriptional regulation. The overall structure of the human TOP2B core dimer adopts a more open quaternary conformation than in other non-human DNA-bound structures reported [9]. The significant changes in the relative dimensional orientation between the main DNA-contacting domains of the two monomers decrease the buried surface area. Etoposide is one of the major inhibitors for TOP2B [9]. Because the active centre of TOP2 is assembled in trans with the catalytic tyrosine (located in the WHD domain) and the Mg^2+^-chelating triad of acidic amino acid residues (E477, D557 and D559; situated in the TOPRIM domain) [10], the increase in distance between the active-site tyrosine and the Mg^2+^-chelating residues suggests that etoposide stabilizes the cleavage complex. If one assumes that etoposide simply traps a pre-existing conformation of the TOP2 cleavage complex, this drug-bound structure may represent a functionally relevant quaternary conformation. The structure thus represents a putative intermediate between a closed post cleavage state [11,12] and the open conformation [13]. The quinoline scaffold exhibited DNA TOP2 inhibition [14], and polycyclic heteroaromatic systems which incorporates a quinoline moiety fused with another heterocycle demonstrated cytotoxicity against a large number of cancer cells [15–17]. Moreover, pyranoquinoline alkaloids showed a wide range of biological activities [18]; several of these alkaloids exhibited a selective cytotoxicity profile showing the greatest activity with oestrogen receptor-positive ZR-75-1 breast cancer cells [19]. On the other hand, a wide range of Schiff's bases revealed interesting inhibitory activity against experimental tumour cells. It is also suggested that the Schiff's bases could be hydrolysed selectively by the tumour cells to act as alkylating agents at the same time as the active amine becomes free to act as an antimetabolite [20]. The active centres of cell constituents are supposed to interact with azomethine's nitrogen atom by forming a hydrogen bond which interferes with normal cell processes and results in the destruction of enzymatic activity of cancerous cells, thereby presenting Schiff's bases as a potential target to discovering anti-cancer chemotherapeutics [21]. Based on the above findings, the aim in this research is to develop a novel series of Schiff's bases containing pyrano­quinolinone with potential anti-cancer activity.

## 2. Results and discussion

**Scheme 1.**
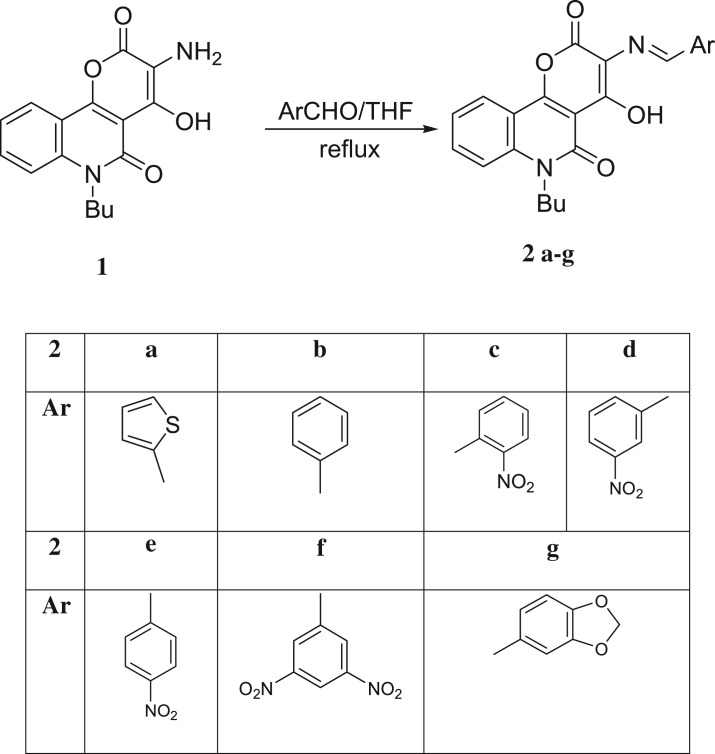
Synthesis of the target compounds.

The 3-aminopyranoquinolinone **1** was prepared by the method reported by Hassanin *et al.* [22]. This amino derivative was condensed with some aromatic aldehydes in dry tetrahydrofuran (THF) to give the target Schiff's bases **2a-g** (scheme 1). The infrared (IR) spectra of compounds **2a-g** show a newly stretching vibration absorption band around 1600–1630 cm^−1^ attributed to (HC=N), in addition to the absence of the double stretching absorption bands of the amino group. Each ^1^H-nuclear magnetic resonance (NMR) spectrum of the synthesized Schiff bases exhibits a singlet signal in a region at 9.09–9.94 ppm attributed to a CH=N- proton. The ^13^C-NMR spectra provide further support for the structural characterization of the Schiff bases. The number of signals found corresponds with the presence of magnetically non-equivalent carbon atoms. All the proposed structures were confirmed by liquid chromatography-mass spectrometry (LC-MS) or electrospray ionization-mass spectrometry (ESI-MS) techniques.

## 3. Molecular modelling study and biological evaluation

**Figure 1.**
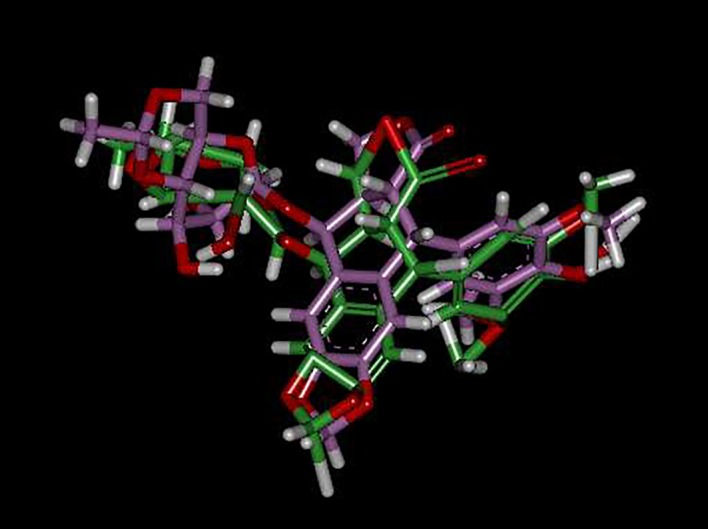
Alignment of bioactive conformer of etoposide lead compound from PDB in green and the best docked conformer in pink.

A molecular modelling study was initiated in order to support the assumed mode of action for tested compounds and optimize a reliable model for predicating novel effective antitumour hits. A docking study was carried out for the target compounds into TOP2B using the Discovery Studio 2.5 software (Accelrys Inc., San Diego, CA, USA). X-ray crystal structure of TOP2B with the ligand molecule etoposide (3QX3) was obtained from protein data bank PDB [23]. The prepared protein was used in determination of the important amino acids in the ATP-binding pocket. Interactive docking using the genetic optimization for ligand docking (GOLD) protocol was carried out for all the conformers of each compound of the test set (**2a-g**) to the selected active site, after energy minimization using the prepared ligand protocol. Re-docking lead compounds with the same binding site showed docking energy =−22.4 kcal mol^−1^ with small root mean square deviation (RMSD) (0.904 A) in comparison to its crystal structure. The small RMSD values proved the validity of the used docking processes (figure 1).

**Figure 2.**
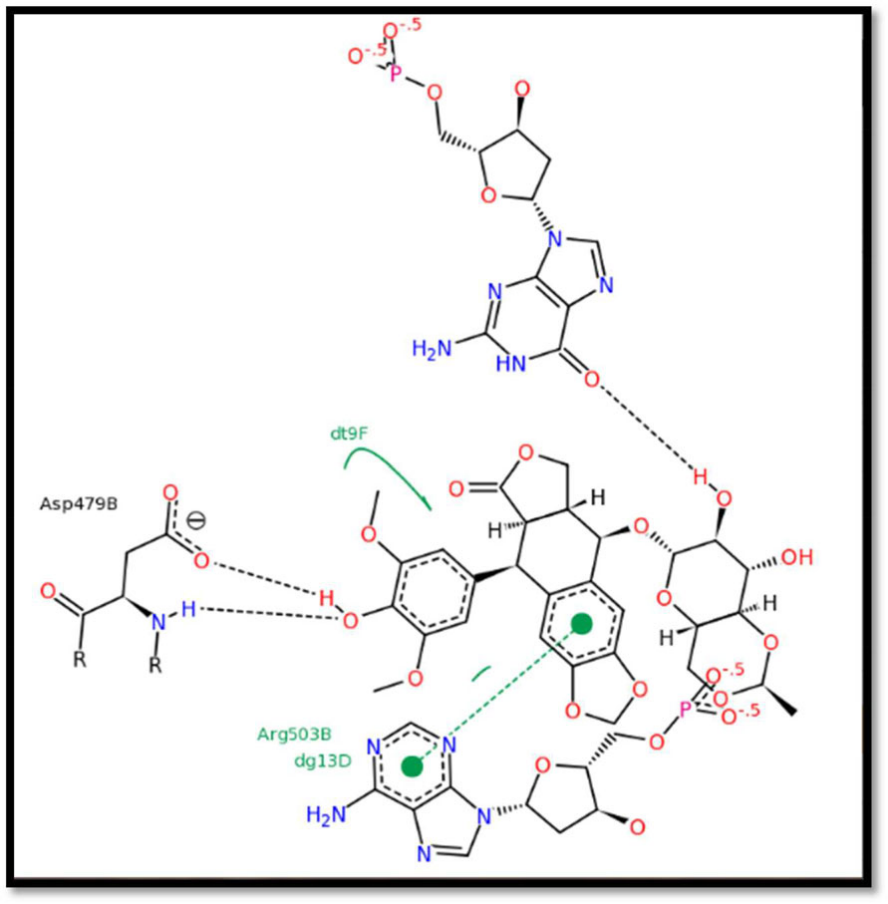
Reported etoposide-binding mode in the active site of TOP2B enzyme.

Each docked compound was assigned a score according to its mode of binding onto the binding site that predicted binding energies and the corresponding experimental values as outlined in table 2. The molecular docking simulation study revealed that the crucial residues of the main hinge region are Asp479 and Arg503, which are thought to be important in explaining the binding behaviour of etoposide toTOP2B as reported [14,15] (figure 2).

**Figure 3:**
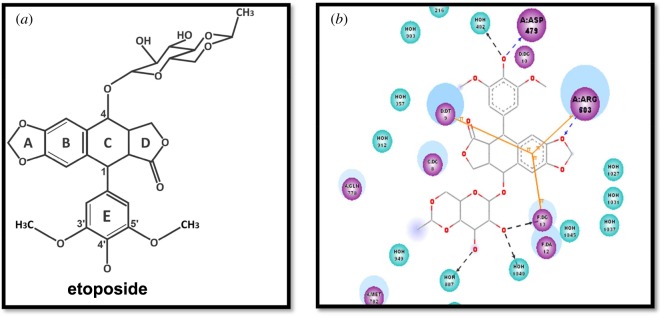
(*a*) Structure of etoposide and (*b*) two-dimensional binding mode of etoposide compound in the constructed active site of TOP2B.

The etoposide lead binding mode indicated that the aromatic ring of the tetracyclic structure makes three π-interactions with different residues including Arg503, while the phenolic oxygen in the para position of the benzene ring is involved in H-bonding with Asp479 in the groove of the TOP2B binding site, while the extended chains are solvated and occupy a hydrophobic pocket. Further validation of our constructed active site of TOP2B was done by docking the etoposide molecule into the active site of and examining the binding mode. The binding mode of etoposide was matched with the reported data as shown in figure 3.

The binding behaviour of the molecule was analysed by docking of the pharmacologically active molecule into the active site of the protein. The residue involved in making the hydrogen bonds is Asp479 and that involved in pi interaction is Arg503 in the hinge region in the binding pocket. All interaction behaviours, as obtained from the docking studies, were found to be in accordance with the reported binding mode of the etoposide in the active site of TOP2B [23,24]. The active compounds adopted the same bioactive conformation of the lead compound and retained the same binding mode of the lead with higher interaction energy, and hence illustrated comparable biological activity which was verified by biological assay. The TOP2B inhibition per cent of the synthesized compounds and their docking scores are given in tables 1 and 2.

**Table 1.** The per cent inhibition of the test set on TOP2B.

compound				results
				MCF-7
ser	cpd. code.	M.Wt g mol^−1^	conc. (μM)	TOP2B %inhibition
**1**	**2a**	394	10	82.36
**2**	**2b**	388	10	60.67
**3**	**2c**	433	10	78.22
**4**	**2d**	433	10	67.098
**5**	**2e**	433	10	66.83
**6**	**2f**	478	10	78.65
**7**	**2g**	432	10	70.15
**8**	ref. (doxorubicin)	579.98	10	74.18

**Table 2.** Inhibition per cent of TOP2B and GOLD docking score for each compound in the test set.

compound	etoposide	2a	2b	2c	2d	2e	2f	2g
% of inhibition	—	82.36	60.67	78.22	67.098	66.83	78.65	70.15
GOLD score (-Kcal mol^−1^)	22.4	29.98	18.97	26.98	20.94	20.02	28.11	21.28

**Figure 4.**
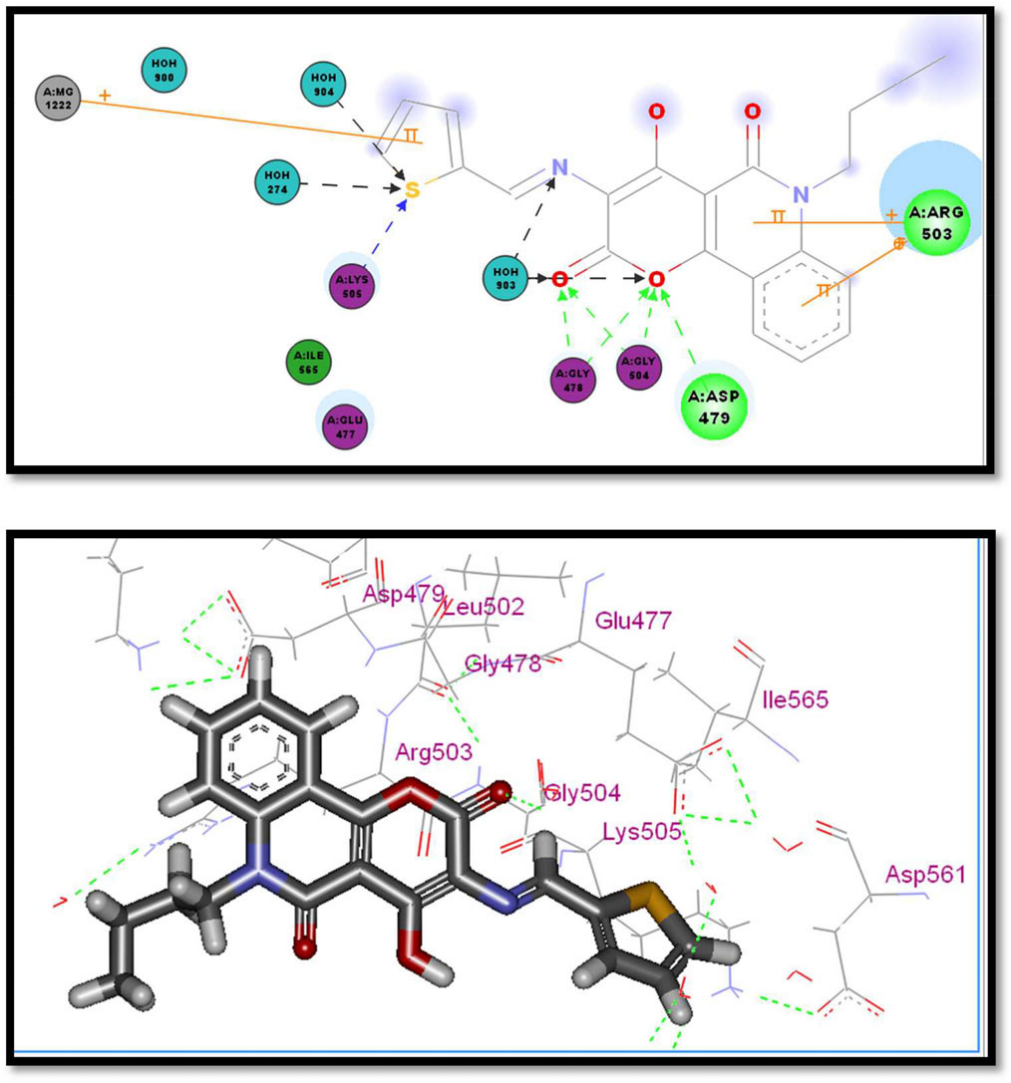
Two- and three-dimensional interaction of compound **2a** in the active site.

**Figure 5.**
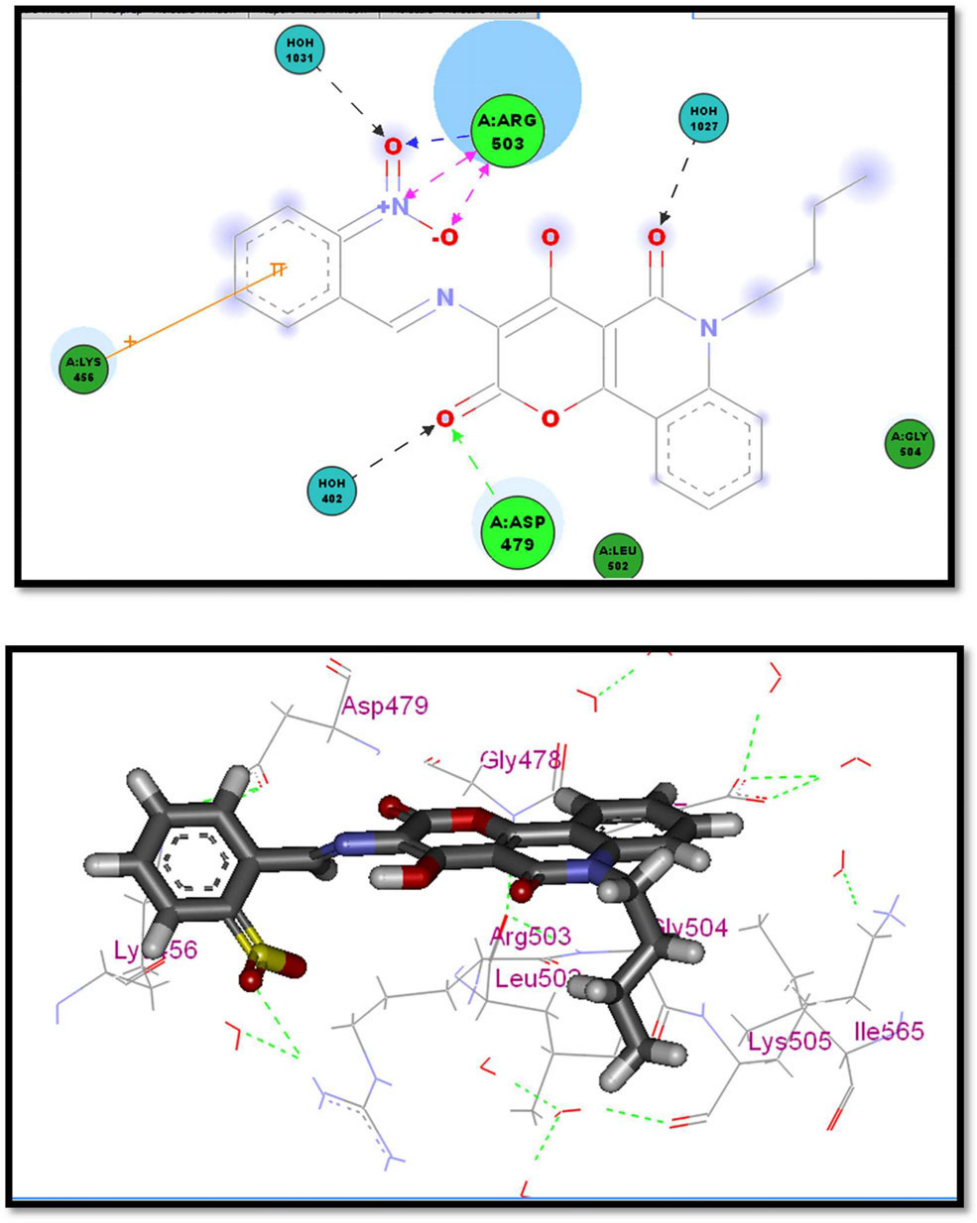
Two- and three-dimensional interaction of compound **2c** in the active site.

**Figure 6.**
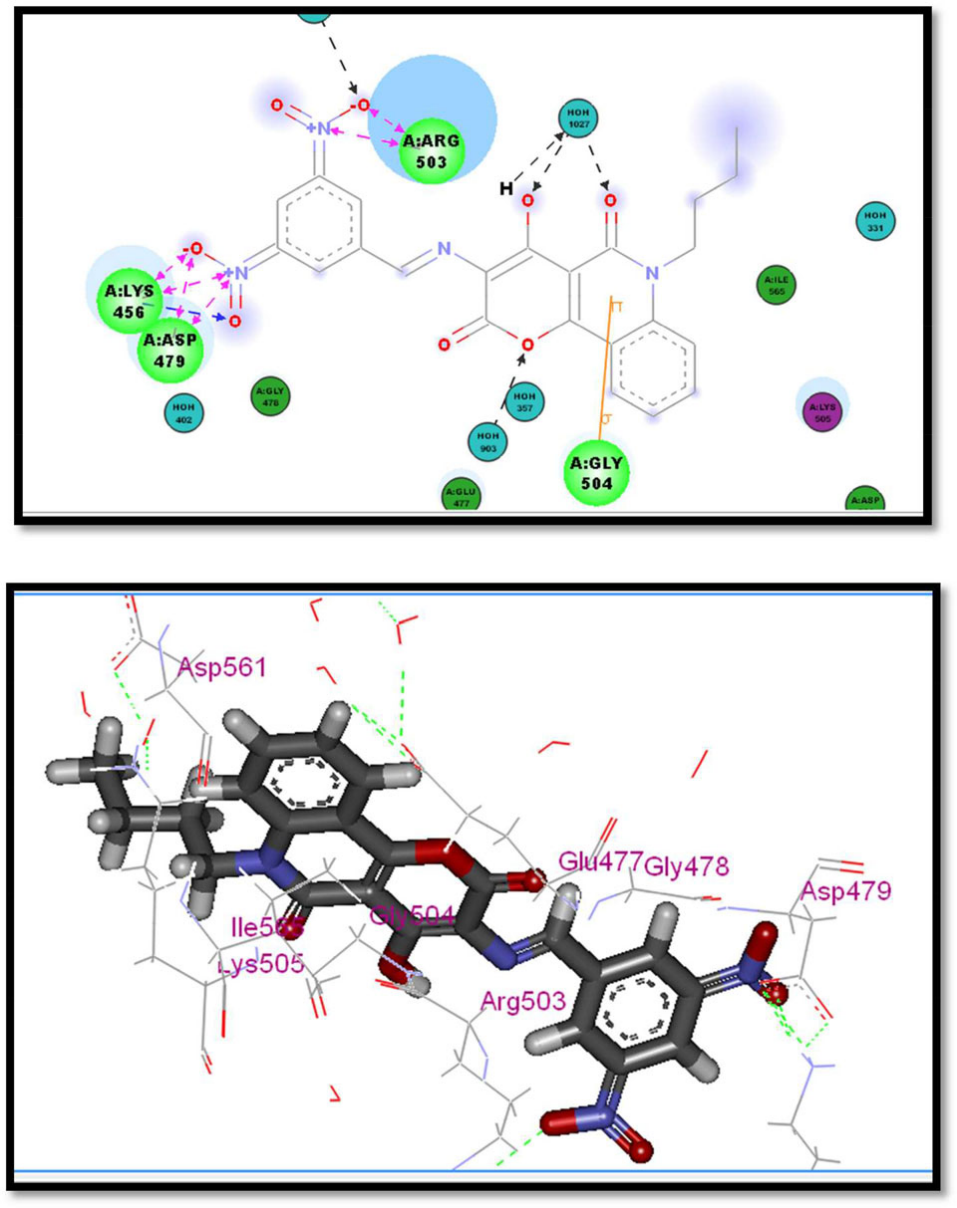
Two- and three-dimensional interaction of compound **2f** in the active site.

Compounds **2a**, **2c** and **2f** which showed a similar binding mode to the lead compound with high docking score values (82.36% −29.98 kcal mol^−1^ for compound **2a**, 78.18%− 26.98 kcal mol^−1^ for compound **2c** and 78.65, −28.11 kcal mol^−1^ for compound **2f**) revealed the highest enzyme inhibition activity (figures 4–6).

The general binding mode of the active hits that showed high inhibition per cent indicated that the carbonyl group in the active hits conserved the H-bond interaction with Asp479 in the hinge region and the butyl group extended chain occupied the hydrophobic pocket and solvated to form extra hydrogen bonding with more amino acids in the pocket region, which stabilizes the active conformer in addition to the chelating bond with Mg^++^in case of compound **2a**, which showed higher inhibition than the reference compound doxorubicin (82.36 versus 74.18). The magnesium ions form salt bridges with both the protein and the DNA. Moreover, compound **2c** which also showed higher inhibition per cent than the reference compound doxorubicin (78.22 versus 74.18) formed many hydrogen bonds with Arg503 which are much stronger than the pi interactions made by etoposide, which also is expected to highly stabilize the active conformer in the binding pocket. Interestingly, the results of compound **2f** support the idea of the importance of the nitro group as an essential pharmacophoric feature. The two nitro moieties form multiple hydrogen bondings with the two crucial amino acids Asp479 and Arg503 required, which gain a compound potent activity of 78.65 versus 74.18. This information may aid to help explain the highly potent activity of compounds **2a**, **2c** and **2f**.

Other amino acids are also involved in binding which are also reported to be essential for activity. For example compound **2a** which possessed the highest activity also made hydrogen bonds with Gly504, Gly478 and Lys505, as shown in figure 5. This information may contribute to explaining the highly potent activity of compound **2a**. It was observed that some of the compounds having moderate inhibition activity like compound **2 g** (70.15%) although having high GOLD score fitness lacked the crucial interaction with Asp479, which may explain the reduction in activity as shown in figure 5.

**Figure 7.**
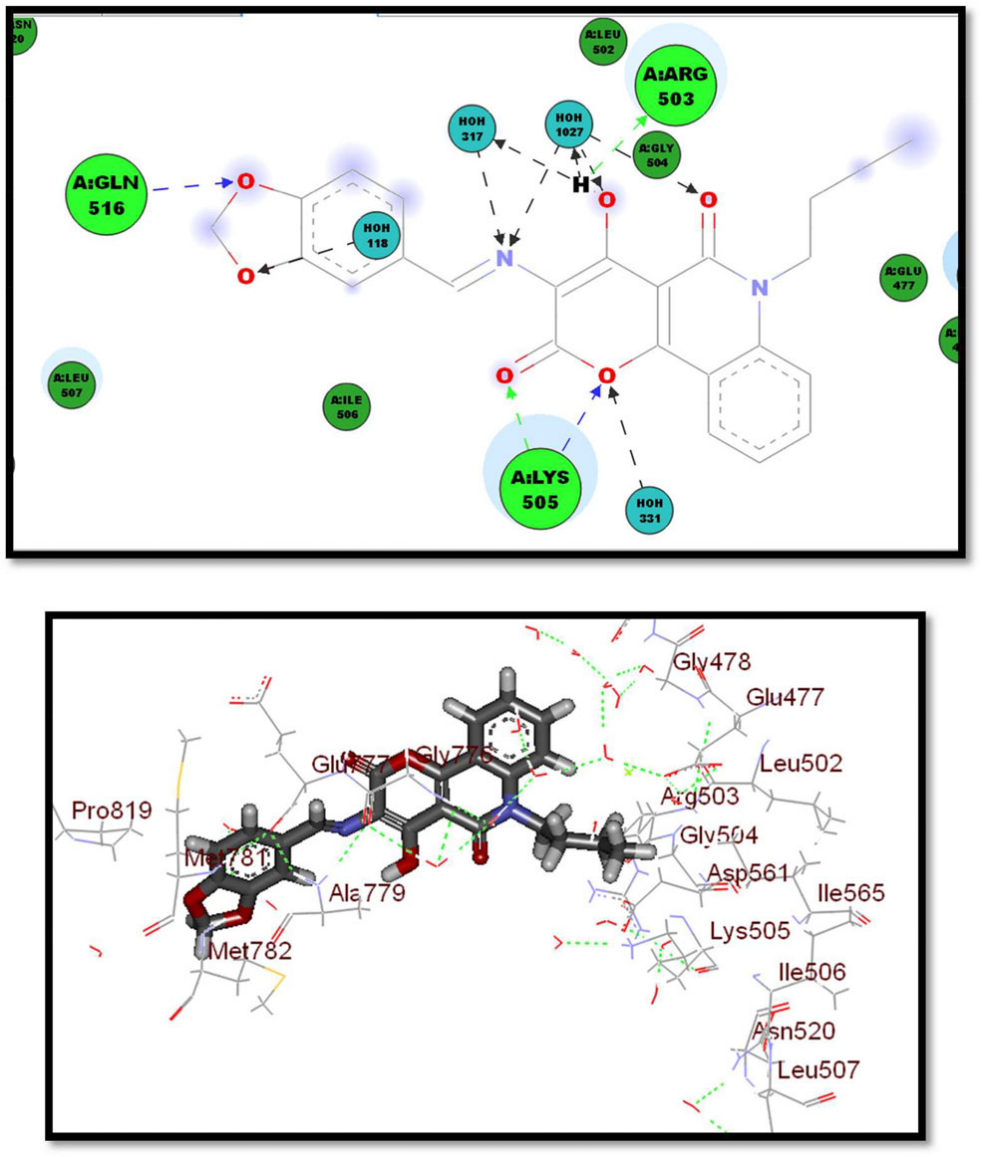
Two- and three-dimensional interaction of compound **2g** in the active site.

**Figure 8.**
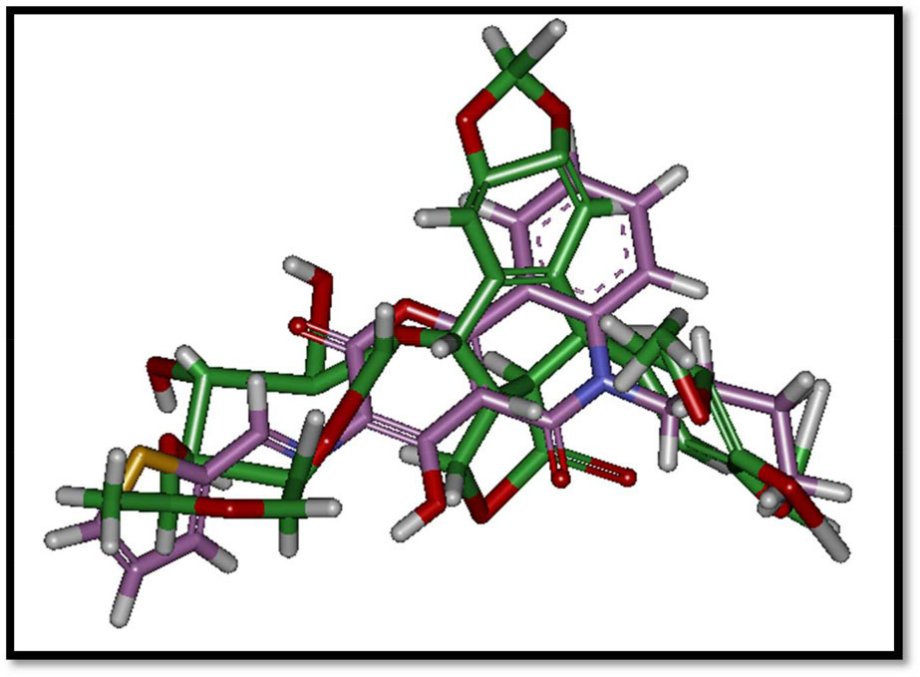
Alignment of compound **2a** (with pink colour) and the lead compound (with green colour) in the active site.

These observed features support the attained SAR assumptions owing to enzymatic inhibitory activity data of the prepared compounds, which could be considered promising hits for developing good effective leads (figures 7 and 8).

To attain more information about the biological activity of the best hit compounds, the IC50 for each compound was measured and is displayed in table 4.

As seen from table 4, the hit compounds exhibited submicromolar IC50 at 5 µM compared to the reference doxorubicin that makes compounds **2a**, **2c** and **2f** highly potent TOP2B cytotoxicity and can be further investigated in clinical trials as potential anti-cancer agents.

## 4. Conclusion

In this paper, a series of novel pyranoquinolinone based Schiff's bases were synthesized and evaluated for TOP2B inhibitory activity and cytotoxicity against breast cancer cell line (MCF-7). Most of the compounds exhibited significant TOP2B inhibitory activity at 10 µM concentration compared to the reference doxorubicin. A molecular docking study was employed to investigate their binding and functional properties as TOP2B inhibitors. Compounds **2a**, **2c** and **2f** showed similar binding mode to the lead compound with high docking score values (82.36% −29.98 kcal mol^−1^ for compound **2a**, 78.18% −26.98 kcal mol^−1^ for compound **2c** and 78.65, −28.11 kcal mol^−1^ for compound **2f**) and revealed higher inhibition per cent than the reference compound doxorubicin. Compounds **2a**, **2c** and **2f** displayed significant TOP2B cytotoxicity in low micromolar range 5 µM compared to the reference doxorubicin. The active compounds adopted the same bioactive conformation of the lead compound and retained the same binding mode of the lead with higher interaction energy and hence illustrated comparable biological activity which was verified by biological assay. Considering the previous results, we have concluded that we have three compounds **2a**, **2c** and **2f** that can be further investigated in clinical trials as potential anticancer agents.

## 5. Experimental set-up

### 5.1. General

Thin layer chromatography (TLC) analysis of the reaction mixtures was performed using Fluka analytical silica gel 60 F254 nm TLC plates. Melting points were recorded on a Sanyo Gallenkamp MPD 350-BM 3.5 Melting Point apparatus. A Thermo Nicolet Nexus 470 Ft-IR spectrophotometer was used for IR analyses. ^1^H-NMR (400 MHz) and ^13^C-NMR (101 MHz) measurements were performed using a Varian-400 MHz spectrometer, and chemical shifts are expressed in *δ* (ppm) relative to tetramethylsilane (in CDCl_3_ or DMSO-*d*_6_ as solvent) as the internal standard. Elemental microanalyses were performed on a Perkin-Elmer CHN-2400II at the Chemical War Department, Ministry of Defence, Cairo, Egypt. Mass spectra were recorded on a Gas Chromatographic GCMSqp 1000 ex Shimadzu instrument at 70 ev., a triple–quadruple tandem mass spectrometer (Micromass W Quattro micro^TM^, Waters Corp., Milford, MA, USA) or a Waters ZMD Quadrupole equipped with ESI.

### 5.2. General procedure for preparation of Schiff bases **2a-g**

A mixture of compound **1** (3 g, 10 mmol) in dry THF (25 ml) and an equivalent amount of the appropriate aldehyde (10 mmol) in dry THF was heated under reflux. The progress of the reaction was monitored by TLC; our target product was precipitated hot. The reaction mixture was filtered and the filtrate was discarded. The solid material obtained was filtered, washed several times by water, dried and crystallized from a suitable solvent to give Schiff bases as illustrated in table 3.

**Table 3.** Schiff bases.

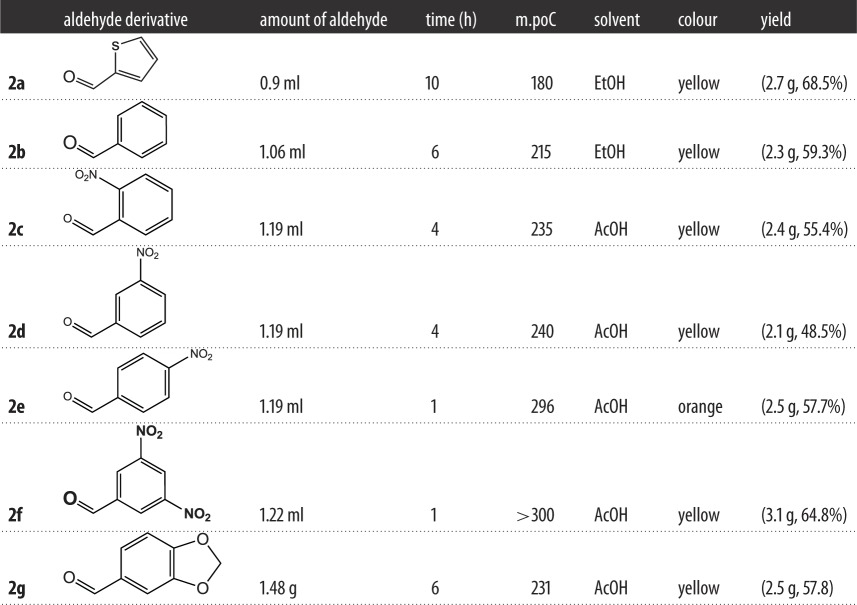

**Table 4.** IC50 of the hit compounds at 5 µM.

compound				results
				MCF-7
ser	cpd. code.	M.Wt g mol^−1^	conc. (μM)	TOP2B -IC50 μM
1	2a	394	5	0.42 ± 0.055
2	2c	433	5	0.83 ± 0.12
3	2f	478	5	0.6 ± 0.07
4	doxorubicin	579.98	5	1.17 ± 0.26

#### 5.2.1. 6-Butyl-4-hydroxy-3-(thiophen-2-ylmethylidene]amino)-2H-pyrano[3,2-c] quinoline-2,5 (6H)-dione (**2a**)

IR (KBr, cm^−1^): 3415 broad band (OH), 3076 (CH_aromatic_), 2948, 2918, 2860 (CH_aliphatic._), 1714 (C=O*_α-_*_pyrone_), and 1668 (C=O_quinoline_), 1609 (C=N), 1598 (C=C_aromatic_). ^1^H NMR(400 MHz, CDCl_3_) *δ* ppm 1.01 (t, *J *= 8.00 Hz, 3 H,C4′, CH_3_), 1.43–1.57 (m, 2 H,C3′,CH_2_), 1.73–1.79 (m, 2 H,C2′, CH_2_), 4.34 (t, *J *= 8.00 Hz, 2 H,C1′, NH-CH_2_), 7.21 (t, *J *= 8.00 Hz, 1 H, C9-H), 7.40 (t, *J *= 8.00 Hz, 1 H_thiophene_), 7.45 (d, *J *= 8.00 Hz, 1 H, C7-H), 7.66 (t, *J *= 8.00 Hz, 1 H,C8-H), 7.75–7.81 (m, 2 H_thiophene_), 8.26 (dd, *J *= 8.00, 0.8 Hz, 1 H, C10-H) 9.94 (s, 1 H, CH=N), 12.39 (s, 1 H, C4-OHexchangeable in D_2_O).^13^C NMR (101 MHz, CDCl_3_) *δ* ppm: 13.77 (s, C4′), 20.19 (s, C3′), 29.62 (s, C2′), 42.28 (s, C 1′), 101.52 (s, C3), 113.95 (s, C4a), 114.91 (s, C10a), 115.23 (s, C7), 123.60 (s, C10), 124.30 (s, C9), 129.15 (s, C_thiophene_), 130.47 (s, C_thiophene_), 131.82 (s, C8), 136.40 (s, C 6a), 139.99 (s, C_thiophene_), 143.02 (s, C_thiophene_), 146.28 (s, C 10b), 150.01 (s, C 2), 162.82 (s, C 4), 163.25 (s, C, C=N) 163.58 (s, C5). MS, *m/z* (*I_r_*%): 395(M^+^+1; 25), 394 (M^+^; 100). M.F: C_21_H_18_N_2_O_4_S, analysis calculated for C_21_H_18_N_2_O_4_S (394): C, 63.95%; H, 4.60%; N, 7.10%; S, 8.13%. Found: C, 63.90%; H, 4.58%; N, 7.04%; S, 8.11%.

#### 5.2.2. 6-Butyl-4-hydroxy-3-((benzylidene)amino)-*2H*-pyrano[3,2-*c*] quinoline-2,5 (*6H*)-dione. (**2b**)

IR (KBr, cm^−1^): 3408 broad band (OH), 3016 (CH_aromatic_), 2945, 2864 (CH_aliphatic._), 1715 (C=O*_α_*_-pyrone_), and 1663 (C=O_quinoline_), 1615 (C=N), 1596 (C=C_aromatic_). ^1^H NMR(400 MHz, CDCl_3_) *δ* ppm: 0.99 (t, *J *= 8.00 Hz, 3 H,C4′, CH_3_), 1.41–1.49 (m, 2 H,C3′,CH_2_), 1.70–1.81 (m, 2 H,C2′, CH_2_), 4.44 (t, *J *= 8.00 Hz, 2 H,C1′, NH-CH_2_), 7.34 (t, *J *= 8.00 Hz, 1 H, C9-H), 7.40–7.53 (m, 2H_aromatic_), 7.65–8.02 (m, 4H_aromatic_), 8.28 (dd, *J *= 8.00, 0.8 Hz, 1 H, C10-H), 9.31 (s, 1 H, CH=N), 13.98 (s, 1 H, C4-OHexchangeable in D_2_O).^13^C NMR (101 MHz, CDCl_3_) *δ* ppm: 14.13 (s, C4′), 19.96 (s, C3′), 33.79 (s, C2′), 42.28 (s, C 1′), 100.89 (s, C3), 112.44 (s, C4a), 114.03 (s, C10a), 115.03 (s, C7), 116.37 (s, C_phenyl_), 119.43 (s, C_phenyl_), 122.71(s, C_phenyl_), 123.26 (s, C10), 124.63 (s, C9), 132.20 (s, C8), 133.35 (s, C_phenyl_), 136.11 (s, C 6a), 136.29 (s, C_phenyl_), 144.47 (s, C_phenyl_), 151.27 (s, C 10b), 152.14 (s, C 2), 159.23 (s, C 4), 162.84 (s, C, C=N), 163.02 (s, C5). MS, *m/z* (*I_r_*%): 389 (M^+^+1; 16), 388 (M^+^; 30), 368 (89), 364 (42), 358 (43), 339 (100). M.F: C_23_H_20_N_2_O_6_, analysis calculated for C_23_H_20_N_2_O_6_ (388.43): C, 71.12%; H, 5.19%; N, 7.21%. Found: C, 71.15%; H, 5.15%; N, 7.19%.

#### 5.2.3. 6-Butyl-4-hydroxy-3-((2-nitrobenzylidene)amino)-2H-pyrano[3,2-c]quinoline-2,5(6H)-dione (**2c**)

IR (KBr, cm^−1^): 3421 broad band (OH), 2985, 2925, 2872 (CH_aliphatic._), 1725(C=O_α‐*pyrone*_), and 1669(C=O_quinoline_), 1613(C=N), 1596(C=C).^1^H NMR (400 MHz, CDCl_3_) *δ* ppm: 1.01 (t, *J *= 8.00 Hz, 3 H, C 4′), 1.43–1.59 (m, 2 H, C 3′), 1.69–1.85 (m, 2 H, C 2′), 4.36 (t, *J *= 8.00 Hz, 2 H, C 2′), 7.45 (t, *J *= 8.00 Hz, 1 H C9-H), 7.50 (d, *J *= 8.61 Hz, 1 HC7-H), 7.56 (t, *J *= 8.00 Hz, 1 H_phenyl_), 7.68 (t, *J *= 8.00 Hz, 1 HC8-H), 7.78 (t, *J *= 8.00 Hz, 1 H_phenyl_), 8.01 (d, *J *= 8.00 Hz, 1 H_phenyl_), 8.31 (dd, *J *= 8.00, 1.6 Hz, 1 H_phenyl_), 8.33 (dd, *J *= 8.00, 1.6 Hz, 1 HC10-H), 9.87 (s, 1 H CH=N), 12.35 (s, 1 H, C4-OH).^13^C NMR (101 MHz, CDCl_3_) *δ* ppm: 13.75 (s, C4′), 20.61 (s, C 3′), 29.57 (s, C2′), 42.53 (s, C1′), 100.28 (s, C3), 113.31 (s, C 4a), 113.72 (s, C10a), 114.92 (s, C 7), 115.19 (s, C_phenyl_), 123.52 (s, C10), 123.83 (s, C9), 124.91 (s, C_phenyl_), 129.62 (s, C_phenyl_), 130.74 (s, C_phenyl_), 131.84 (s, C10), 132.22 (s, C_phenyl_), 133.36 (s, C_phenyl_), 138.06 (s, C6a), 146.23 (s, C10b), 149.23 (s, C2), 157.41 (s, C=N), 162.52 (s, C4), 163.19 (s, C5). ESI-MS *m/z*: 434.7 [M + H]^+^, 435.2 [M + 2H]^+^, 456.3 [M + Na]^+^. M.F: C_23_H_19_N_3_O_6_, analysis calculated for C_23_H_19_N_3_O_6_ (433.42): C, 63.74%; H, 4.42%; N, 9.69%. Found: C, 63.70%; H, 4.45%; N, 9.64%.

#### 5.2.4. 6-Butyl-4-hydroxy-3-((3-nitrobenzylidene)amino)-2H-pyrano[3,2-c]quinoline-2,5(6H)-dione (**2d**)

IR (KBr, cm^−1^): 3403 broad band (OH), 3082 (CH_aromatic_), 2959, 2926, 2869 (CH_aliphatic_), 1715(C=O*_α_*_-pyrone_), and 1663(C=O_quinoline_), 1611(C=N), 1563(C=C_aromatic_). ^1^H NMR (400 MHz, DMSO-*d*_6_) *δ* ppm: 0.92 (t, *J *= 8.00 Hz, 3 H,C 4′), 1.38–1.46 (m, 2 H,C 3′), 1.60–1.69 (m, 2 H, C 2′), 4.33 (t, *J *= 8.00 Hz, 2 H,C 2′), 7.50 (t, *J *= 8.00 Hz, 1 HC9-H), 7.74 (d, *J *= 8.00 Hz, 1 HC7-H), 7.82 (d, *J *= 8.00 Hz, 1 H_phenyl_), 7.87 (d, *J *= 8.00 Hz, 1 H_phenyl_), 8.15 (t, *J *= 8.00 Hz, 1 HC8-H), 8.27 (dd, *J *= 8.00, 1.76 Hz, 1 H_phenyl_), 8.65 (dd, *J *= 8.00, 1.76 Hz, 1 C10-H), 9.44 (s, 1 HCH = N), 11.94 (s, 1 H, C 4OH). ^13^C NMR(101 MHz, CDCl_3_) *δ* ppm: 13.72 (s, C4′), 20.16 (s, C3′), 29.57 (s, C 2′), 42.54 (s, C1′), 100.39 (s, C3), 112.69 (s, C4a), 113.76 (s, C10a), 115.18 (s, C7), 123.36 (s, C10), 124.21 (s, C9), 124.93 (s, C8), 125.11 (s, C_phenyl_), 129.52 (s, C_phenyl_), 133.83 (s, C_phenyl_), 134.09 (s, C_phenyl_), 138.13 (s, C6a), 139.18 (s, C_phenyl_), 148.64 (s, C10b), 156.65 (s, C_phenyl_), 157.42 (s, C2), 157.79 (s, C=N), 162.97 (s, C4), 163.21 (s, C5). ESI-MS *m/z*: 434.286 [M + H]^+^, 456.244 [M + Na]^2+^.M.F: C_23_H_19_N_3_O_6_, analysis calculated for C_23_H_19_N_3_O_6_ (433.42): C, 63.74%; H, 4.42%; N, 9.69%. Found: C, 63.70%; H, 4.43%; N, 9.65%.

#### 5.2.5. 6-Butyl-4-hydroxy-3-((4-nitrobenzylidene)amino)-*2H*-pyrano[3,2-c]quinoline-2,5(6H)-dione. (**2e**)

IR (KBr, cm^−1^): 3380 broad band (OH), 3082 (CH_aromatic_), 2959, 2921, 2848 (CH_aliphatic__._), 1722 (C=O_α-*pyrone*_), 1666 (C=O_quinoline_), 1613 (C=N), 1572 (C=C_aromatic_). ^1^H NMR (400 MHz, DMSO-*d*_6_) *δ* ppm: 0.93 (t, *J *= 8.00 Hz, 3 H,C 4′), 1.36–1.43 (m, 2 H,C 3′), 1.60–1.67 (m, 2 H, C 2′), 4.35 (t, *J *= 8.00 Hz, 2 H,C 2′), 7.44 (t, *J *= 8.00 Hz, 1 HC9-H), 7.53 (d, *J *= 8.00 Hz, 1 HC7-H), 7.71 (d, *J *= 8.00 Hz, 1 H_phenyl_), 7.76 (d, *J *= 8.00 Hz, 1 H_phenyl_), 7.78 (t, *J *= 8.00 Hz, 1 HC8-H), 8.01 (dd, *J *= 8.00, 1.76 Hz, 1 H_phenyl_), 8.28 (dd, *J *= 8.00, 1.76 Hz, 1 H_phenyl_), 8.39 (dd, *J *= 8.00, 1.76 Hz, 1 C10-H), 9.48 (s, 1 HCH = N), 11.93 (s, 1 H, C 4OH). ^13^C NMR(101 MHz, CDCl_3_) *δ* ppm: 13.77 (s, C4′), 20.19 (s, C3′), 29.63 (s, 1 C 2′), 42.28 (s, 1 C 1′), 101.59 (s, 1 C 3), 113.95 (s, C4a), 114.91 (s, C10a), 115.23 (s, C7), 123.60 (s, C10), 123.87 (s, C9), 124.30 (s, 2 C_phenyl_), 129.15 (s, C_phenyl_), 130.47 (s, 2 C_phenyl_), 131.82 (s, C8), 136.34 (s, C6a), 143.02 (s, C_phenyl_), 146.28 (s, C10b), 159.32 (s, C4), 162.82 (s, C2), 163.25 (s, C=N), 163.58 (s, C 5). M.F: C_23_H_19_N_3_O_6_, analysis calculated for C_23_H_19_N_3_O_6_ (433.42): C, 63.74%; H, 4.42%; N, 9.69%. Found: C, 63.76%; H, 4.41%; N, 9.66%.

#### 5.2.6. 6-Butyl-4-hydroxy-3-((3,5-dinitrobenzylidene) amino)-*2H*-pyrano [3,2-*c*] quinoline-2,5(*6H*)-dione. (**2f**)

IR (KBr, cm^−1^): 3447, 3292 broad band (OH), 2958, 2932, 2876 (CH_aliphatic__._), 1728 (C=O_α‐pyrone_), 1679(C=O_quinoline_), 1613 (C=N), 1576 (C=C_aromatic_). ^1^H NMR (400 MHz, DMSO-*d*_6_) *δ* ppm: 0.92 (t, *J *= 8.00 Hz, 3 H,C 4′), 1.38–1.43 (m, 2 H,C 3′), 1.63–1.71 (m, 2 H, C 2′), 4.45 (t, *J *= 8.00 Hz, 2 H,C 2′), 7.15 (s, 1 H_phenyl_), 7.45 (t, *J *= 8.00 Hz, 1 HC9-H), 7.53 (d, *J *= 8.00 Hz, 1 HC7-H), 7.76 (s, 1 H_phenyl_), 7.78 (t, *J *= 8.00 Hz, 1 HC8-H), 7.98 (s, 1 H_phenyl_), 8.25 (dd, *J *= 8.00, 1.76 Hz, 1 C10-H), 9.78 (s, 1 HCH = N), 13.53 (s, 1 H, C 4OH). ^13^C NMR(101 MHz, CDCl_3_) *δ* ppm: 14.15 (s, C4′), 19.97 (s, C3′), 29.70 (s, 1 C 2′), 42.29 (s, 1 C 1′), 100.93 (s, 1 C 3), 112.46 (s, C4a), 113.89 (s, C10a), 114.09 (s, C7), 116.43 (s, C_phenyl_), 116.63(s, C_phenyl_), 119.42(s, C_phenyl_),122.75 (s, C10), 123.30 (s, C9), 124.39 (s, C_phenyl_), 124.63 (s, C_phenyl_), 132.18 (s, C8), 133.35 (s, C6a), 136.15 (s, C_phenyl_), 136.97 (s, C10b), 151.25 (s, C4), 152.17 (s, C2), 159.24 (s, C=N), 163.06 (s, C5). ESI-MS *m/z*: 479.5 [M + H]^+^, 480.3 [M + H]^+^, 801.7 [M + Na]^2+^.M.F: C_23_H_18_N_4_O_8_, analysis calculated for C_23_H_18_N_4_O_8_ (478.42): C, 57.74%; H, 3.79%; N, 11.71%. Found: C, 57.70%; H, 3.75%; N, 11.74%.

#### 5.2.7. 3-((Benzo[d][1,3]dioxol-5-ylmethylene)amino)-6-butyl-4-hydroxy-*2H*-pyrano[3,2-*c*]quinoline-2,5(*6H*)-dione.(**2g**)

IR (KBr, cm^−1^): 3408 broad band (OH), 3082 (CH_aromatic_), 2955, 2919, 2872 (CH_aliphatic__._), 1712 (C=O*_α_*_-pyrone_), and 1666 (C=O_quinoline_), 1619 (C=N), 1593 (C=C_aromatic_). ^1^H NMR (400 MHz, DMSO-*d*_6_) *δ* ppm: 0.91 (t, *J *= 8.00 Hz, 3 H,C4′), 1.35–1.45 (m, 2 H,C3′), 1.58–1.68 (m, 2 H, C2′), 4.30 (t, *J *= 8.00 Hz, 2 H,C 1′), 6.09 (s, 2 H_piperonal_), 7.01 (d, *J *= 7.83 Hz, 1 H_piperonal_) 7.33 (dd, *J *= 8.02, 1.37 Hz, 1 H_piperonal_) 7.42 (t, *J *= 8.00 Hz, 1 H C9-H), 7.73 (d, *J *= 7.60 Hz, 1 H C7-H), 7.83 (t, *J *= 7.60 Hz, 1 H C8-H), 8.02 (dd, *J *= 8.02, 1.37 Hz, 1 H_piperonal_) 8.15 (dd, *J *= 8.00, 1.20 Hz, 1 H C10-H), 9.09 (s, 1 HCH = N), 12.27 (s, 1 H, C 4OH).^13^C NMR (101 MHz, DMSO-*d*6) *δ* ppm: 14.14 (s, 4′), 19.94 (s, C3′), 29.71 (s, C2′), 42.08 (s, C1′), 101.75 (s, C3), 102.77 (s,CH_2pipronal_), 106.66 (s, C_pipronal_), 109.01 (s, C_pipronal_), 114.08 (s, C4a), 115.05 (s, C10a), 116.43 (s, C7), 122.73 (s, C10), 124.37 (s, C8), 129.05 (s, C_pipronal_), 131.89 (s, C8), 132.15 (s, C_pipronal_), 136.10 (s, C6a), 144.39 (s, C_pipronal_), 148.84 (s, C10b), 153.19 (s, C_pipronal_), 158.97 (s, C 4), 160.15 (s, C=N), 160.80 (s, C2). 162.85 (s, C5). MS, *m/z* (*I_r_*%): 433 (M^+^+1; 6), 432 (M^+^; 15), 417 (51), 393 (70), 378 (26), 372 (100). M.F:C_24_H_20_N_2_O_6_, analysis calculated for C_24_H_20_N_2_O_6_ (432.44): C, 66.66%; H, 4.66%; N, 6.48%. Found: C, 66.62%; H, 4.64%; N, 6.44%.

## 6. Molecular modelling

All molecular modelling studies were performed using the Accelrys Discovery Studio 2.5 operating system, GOLD protocol at the Faculty of Pharmacy, Ain Shams University, Cairo, Egypt. Molecules were built within DS and conformational models for each compound were generated automatically. Docking study involved the following steps.

The docking analysis was carried out on TOP2B enzyme. The three-dimensional protein structure of TOP2B enzyme co-crystallized with lead etoposide (code; 3QX3) was downloaded from the Protein Data Bank of the Research Collaboration for Structural Bioinformatics (RCSB) website (www.rcsb.org). To evaluate the suitability of the crystal structure and the docking workflow, reference inhibitors were re-docked into the built active site and the RMSD value was calculated and binding interactions were compared to those found in the literature. For all compounds mainly poses inside the binding pocket were generated by the software.

The binding pocket was prepared for docking by cleaning the protein, adding the missing hydrogens and side chains, with energy minimization according to the DS protocol. The binding pocket of the complexed lead compound with the connected amino acid residues was identified at a sphere of radius = 12 Å and then was used in docking of test compounds using the GOLD module. After that the GOLD fitness scores of the best fitted conformation of the docked molecule were recorded (table 2).

## 7. Biological techniques

MC7 cells were obtained from American Type Culture Collection; cells were cultured using RPMI (Invitrogen/Life Technologies) supplemented with 10% fetal bovine serum (Hyclone), 10 µg ml^−1^ of insulin (Sigma) and 1% penicillin–streptomycin. All of the other chemicals and reagents were from Sigma or Invitrogen.

Plate cells (cells density 1.2–1.8 × 10 000 cells well^−1^) were cultured in a volume of 100 µl of complete growth medium + 100 µl of the tested compound per well in a 96-well plate for 18–24 h before the enzyme assay.

Reader: ROBONIK P2000 ELISA READER wl 450 nm
Cell line: MCF-7
Kit used for enzyme-assay: Human DNA Topoisomerase 2B eia Kit MBS942146
Solvent : DMSO
Assay samples: cell culture supernatant

## Supplementary Material

IR, 1HNMR, 13CNMR and mass spectral Analytical data

## Data accessibility

The data of this work are available as the electronic supplementary material. Atomic coordinates and structure factors have been deposited in the Protein DataBank (PDB) under accession code 3QX3.
