# Supplementary material for: Synthesis and molecular docking studies of some novel Schiff bases incorporating 6-butylquinolinedione moiety as potential topoisomerase IIβ inhibitors
Source: R Soc Open Sci. 2018 Jun 20;5(6):172407. doi: 10.1098/rsos.172407 (PMC6030276; doi:10.1098/rsos.172407)
Supplement: IR, 1HNMR, 13CNMR and mass spectral Analytical data [file rsos172407supp1.docx]

**Compound 2a**

**^^**

**IR-spectrum of compound 2a**

**^1^H NMR** **Spectrum of compound no. 2a**

**D_2_O- ^1^H-NMR Spectrum of compound no. 2a**

**^13^C- NMR Spectrum of compound no. 2a**

**
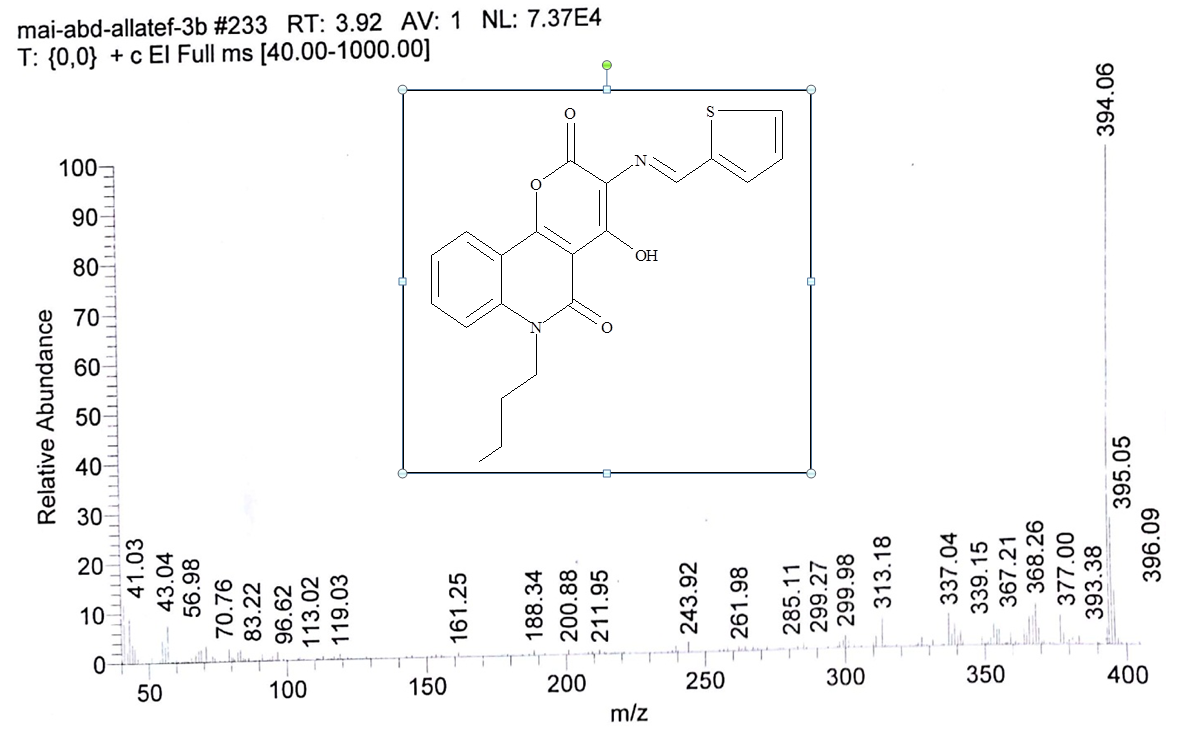
**

**Mass spectrum of compound 2a**

**Compound 2b**

**IR-spectrum of compound 2b**

**
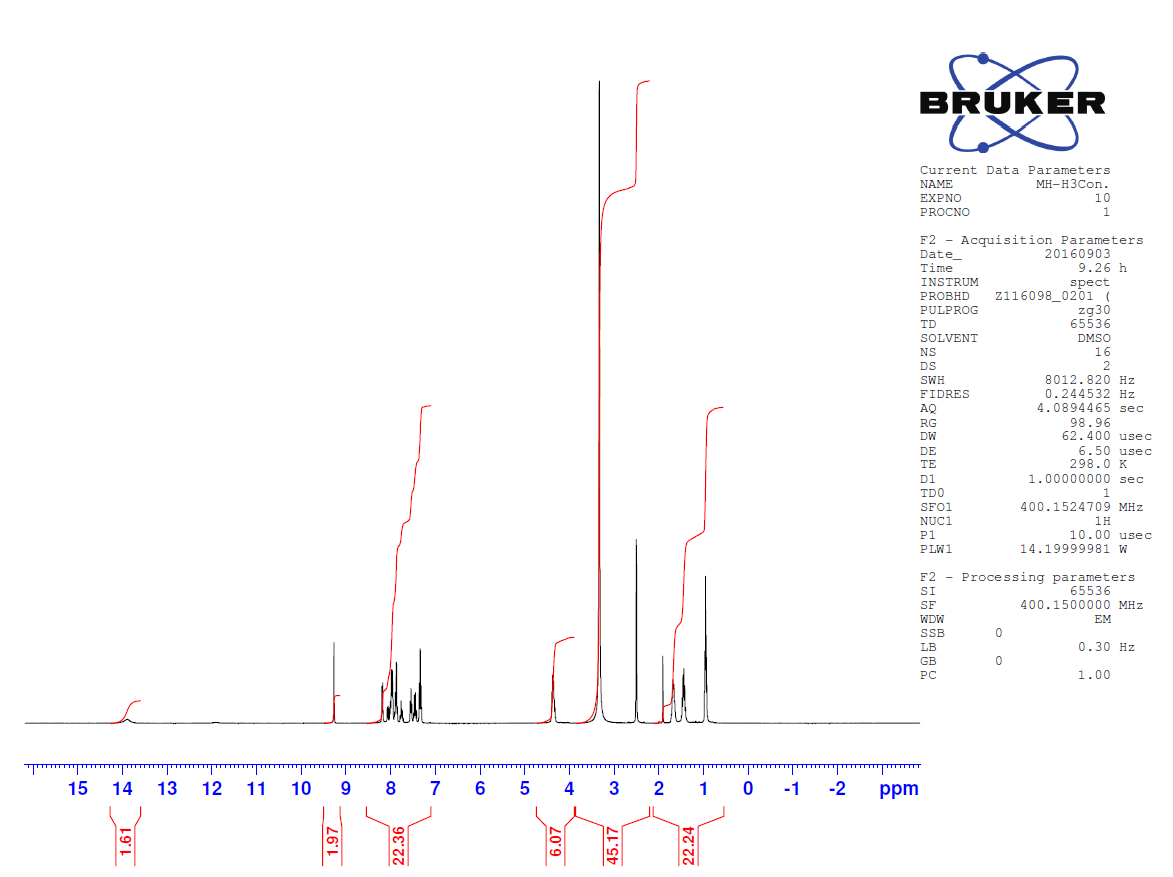
**

**^1^H NMR** **Spectrum of compound no. 2b**

**^
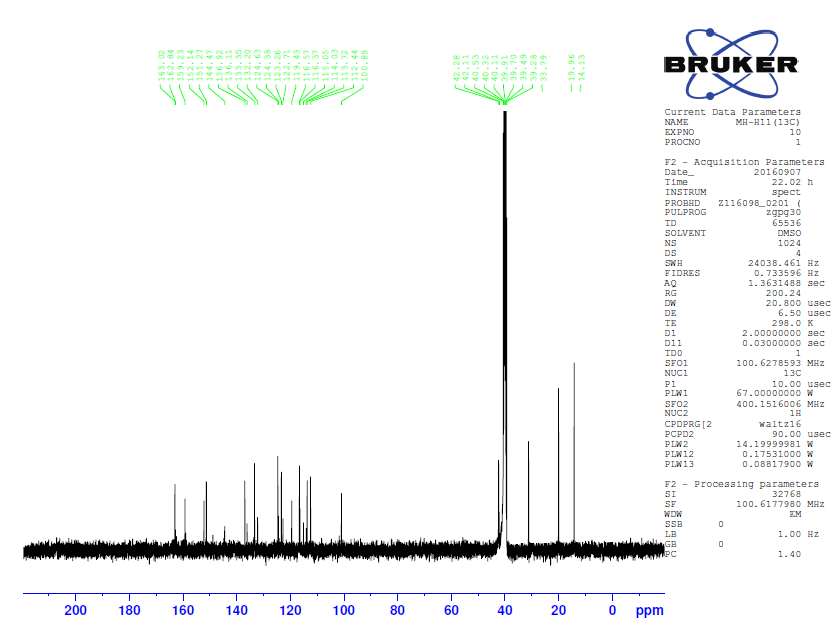
^**

**^13^C NMR** **Spectrum of compound no. 2b**

**Mass spectrum of compound 2b**

**Compound 2c**

**IR-spectrum of compound 2c**

**^1^H NMR** **Spectrum of compound no. 2c**

**^13^C NMR** **Spectrum of compound no. 2c**

**ESI mass Spectrum of compound no. 2c**

**Compound 2d**

**IR spectrum of compound no. 2d**

**^1^H- NMR** **Spectrum of compound no. 2d**

**^13^C- NMR** **Spectrum of compound no. 2d**

**
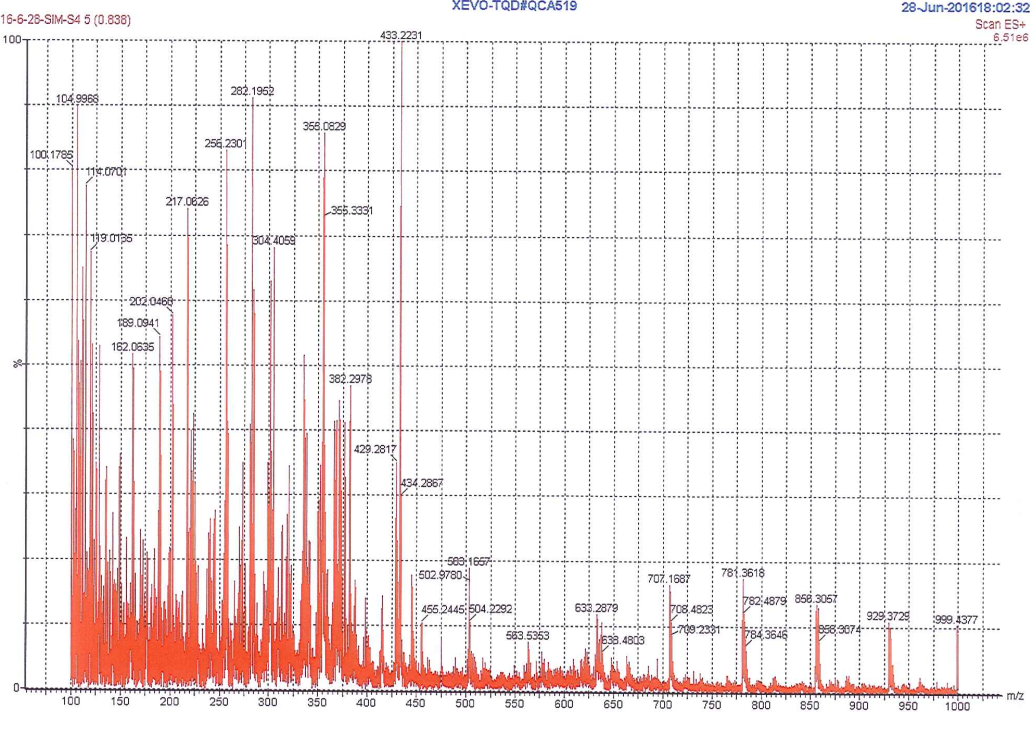
**

**ESI mass Spectrum of compound no. 2c**

**Compound 2e**

**
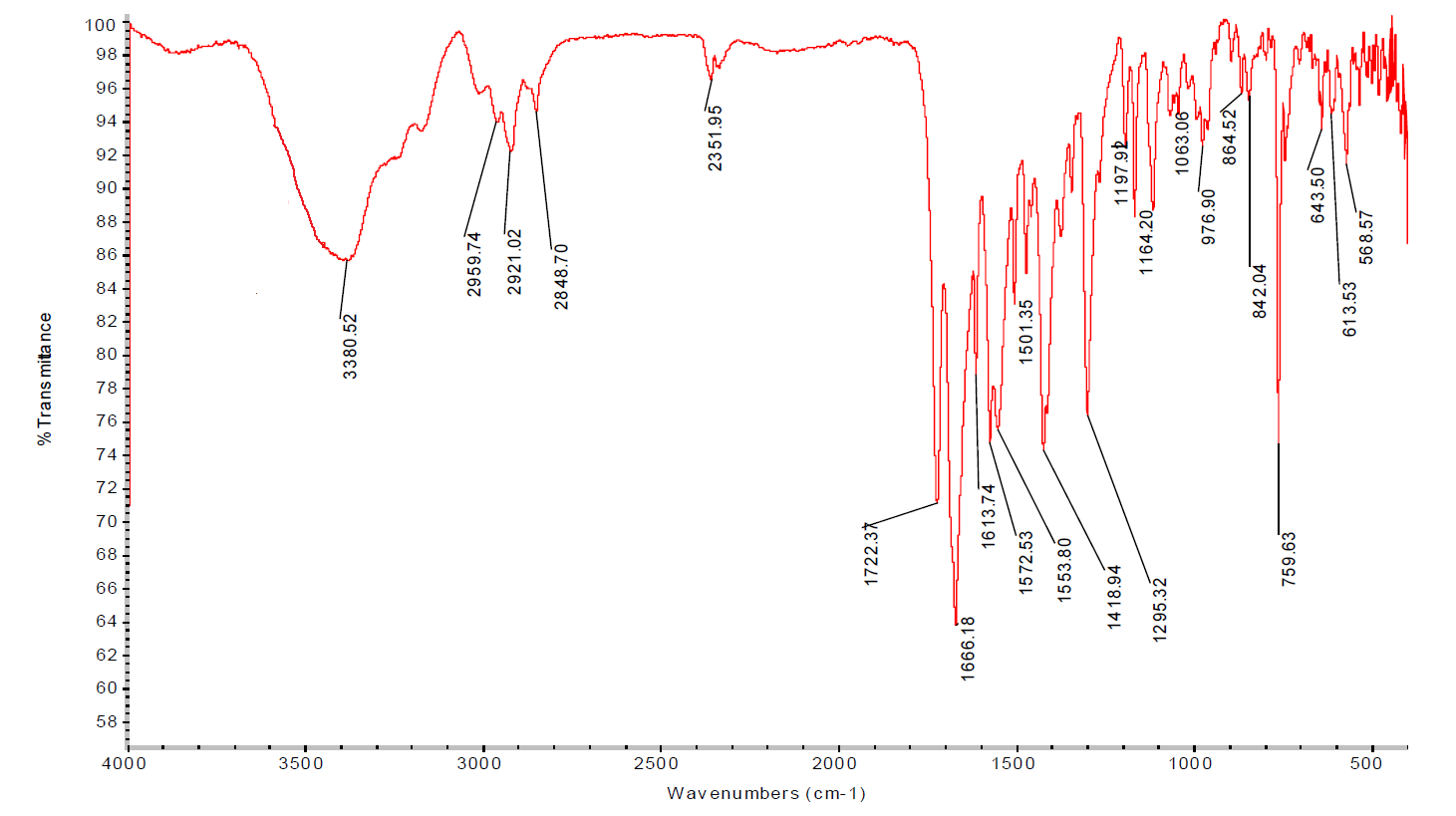
**

**^1^H NMR** **Spectrum of compound no. 2e**

**^13^C NMR** **Spectrum of compound no. 2e**

**Compound 2f**


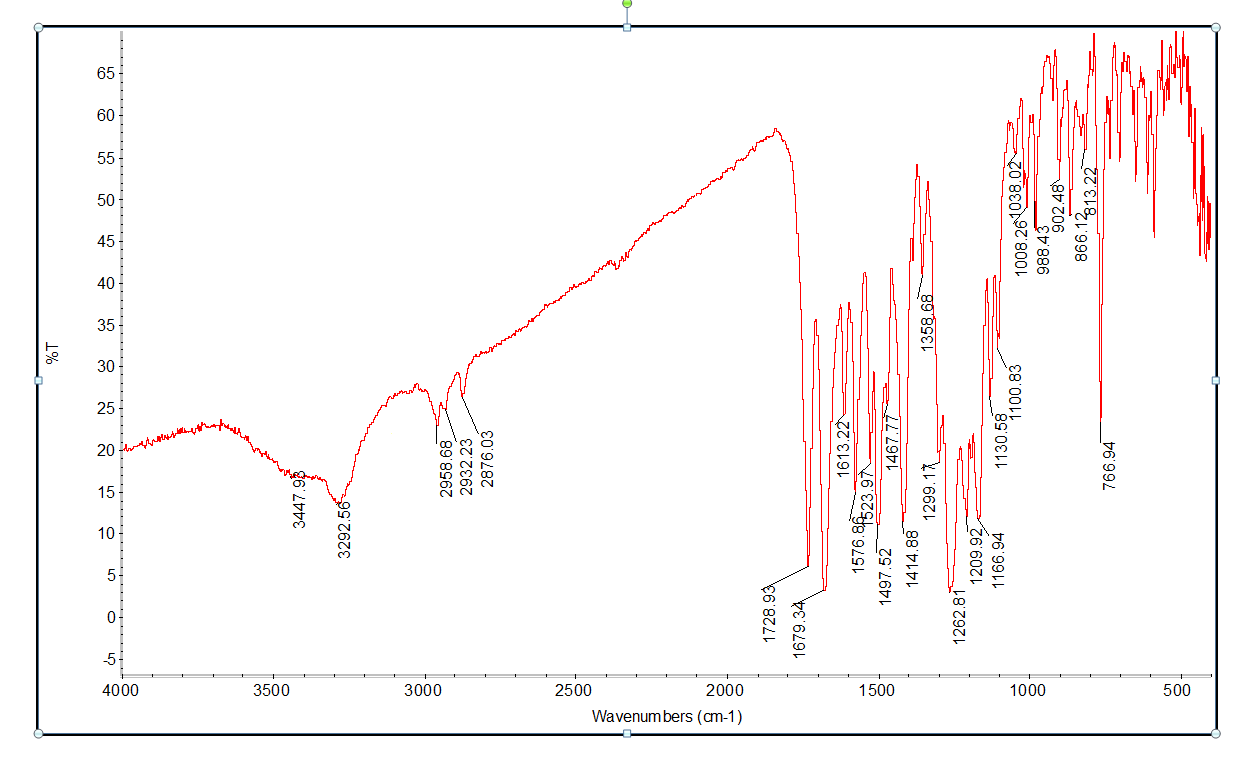


**IR Spectrum of compound no. 2f**


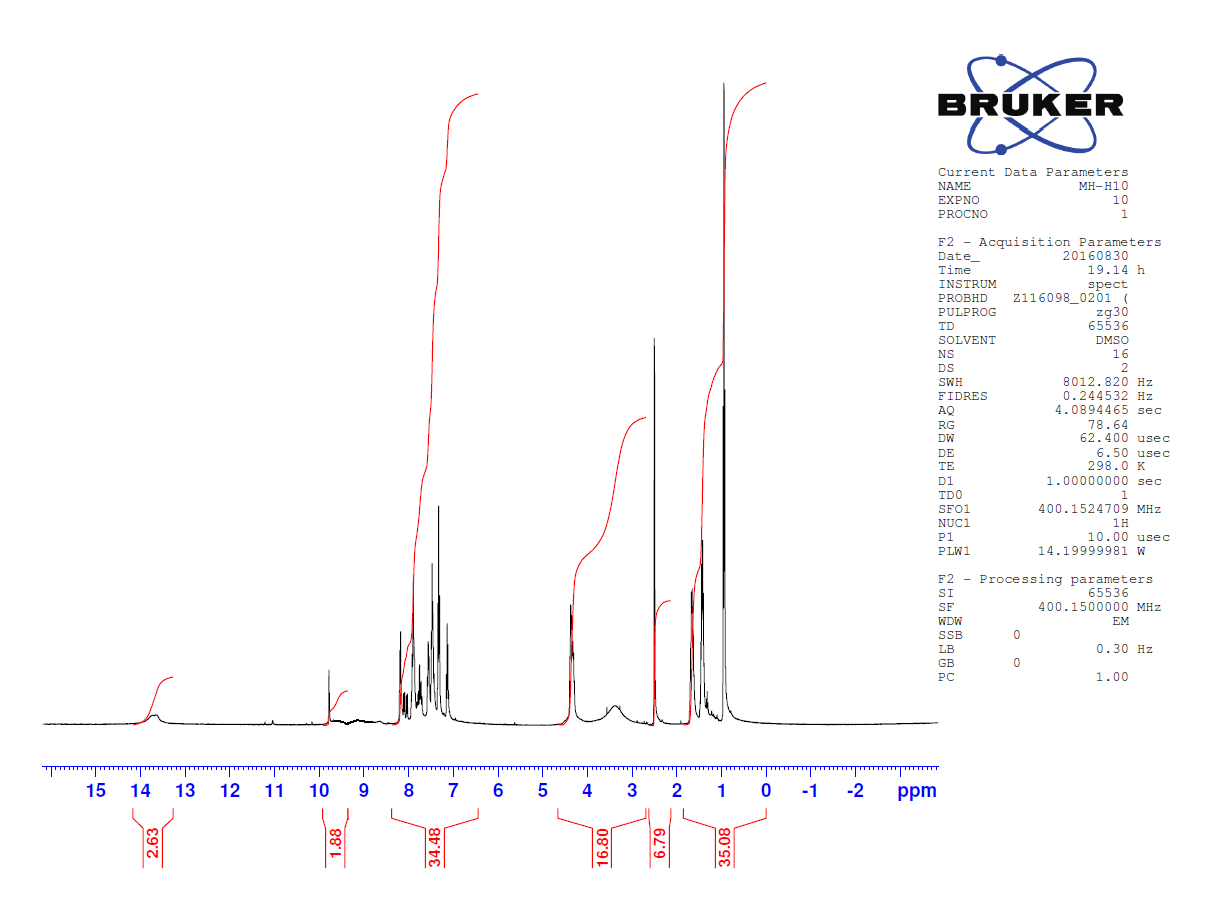


**^1^H NMR** **Spectrum of compound no. 2f**


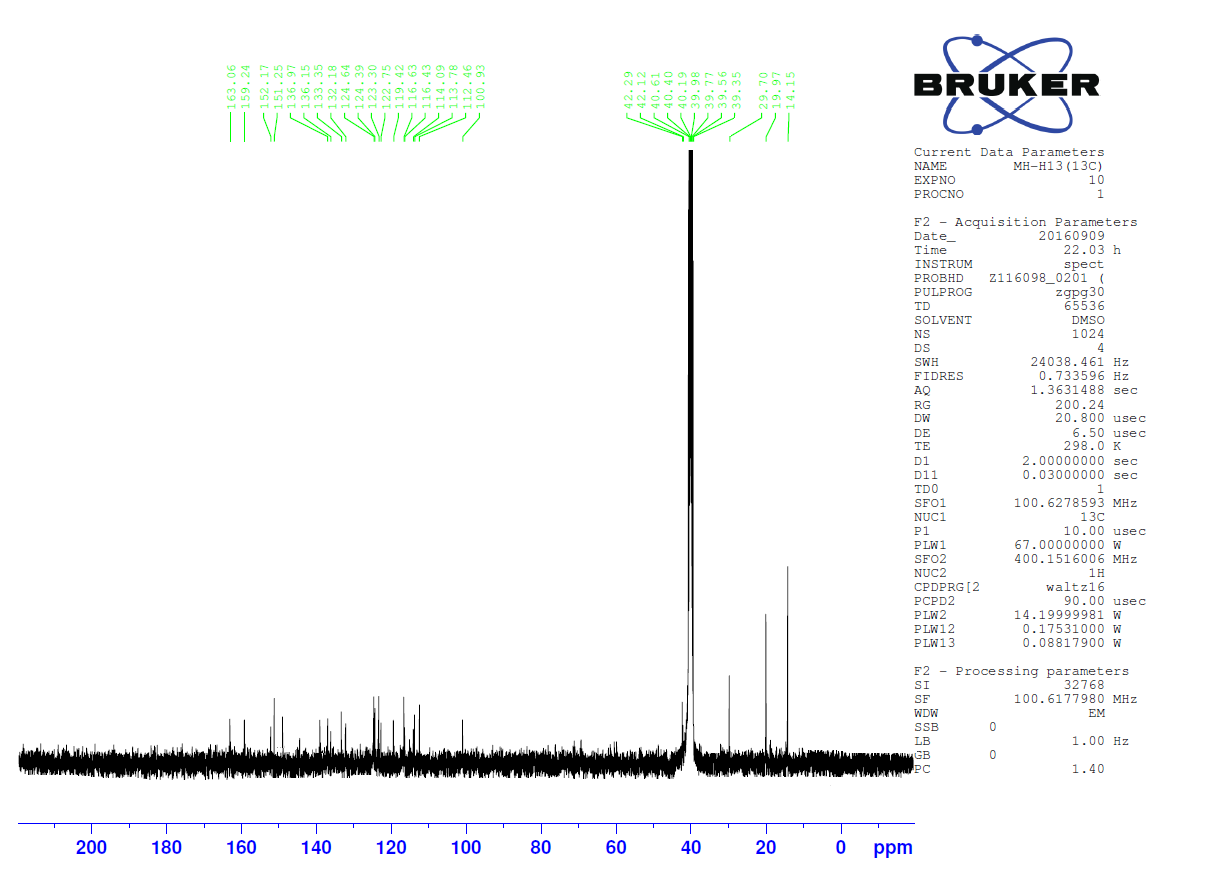


**^13^C NMR** **Spectrum of compound no. 2f**

**ESI mass Spectrum of compound no. 2f**

**Compound 2g**

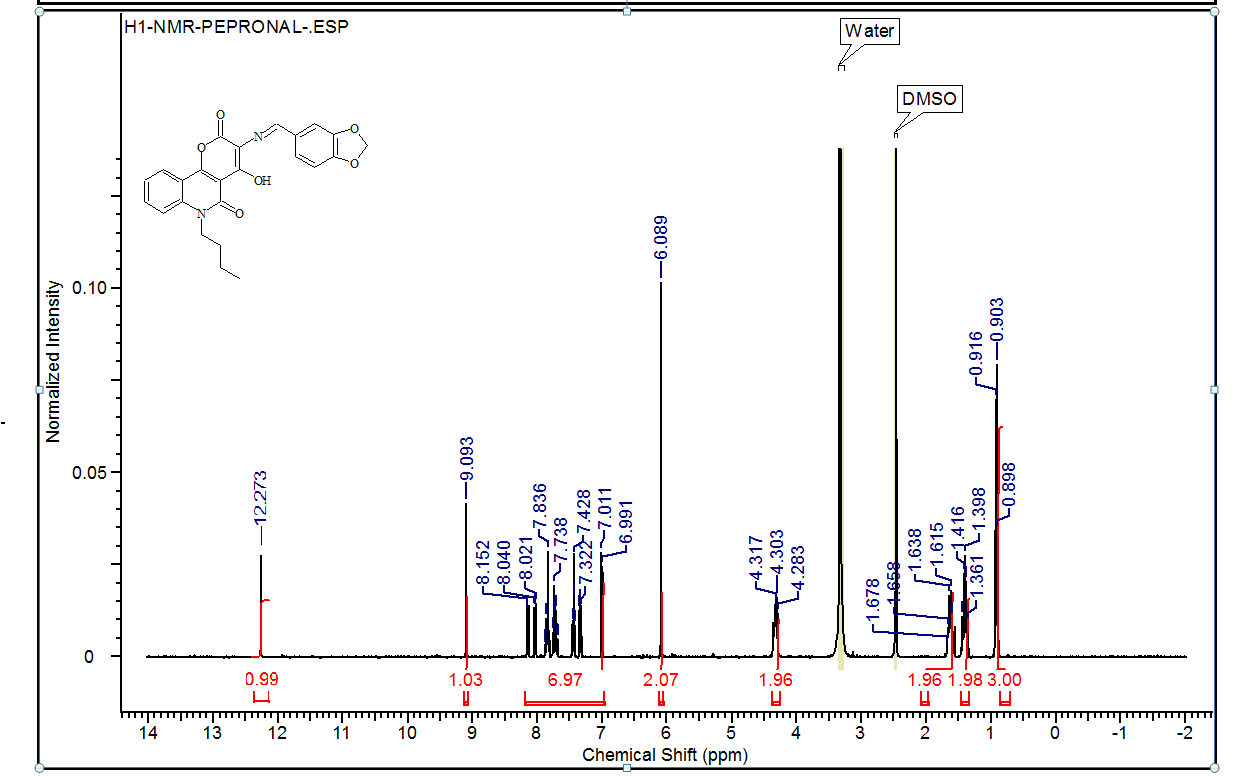


**^1^H NMR** **Spectrum of compound no. 2g**

**
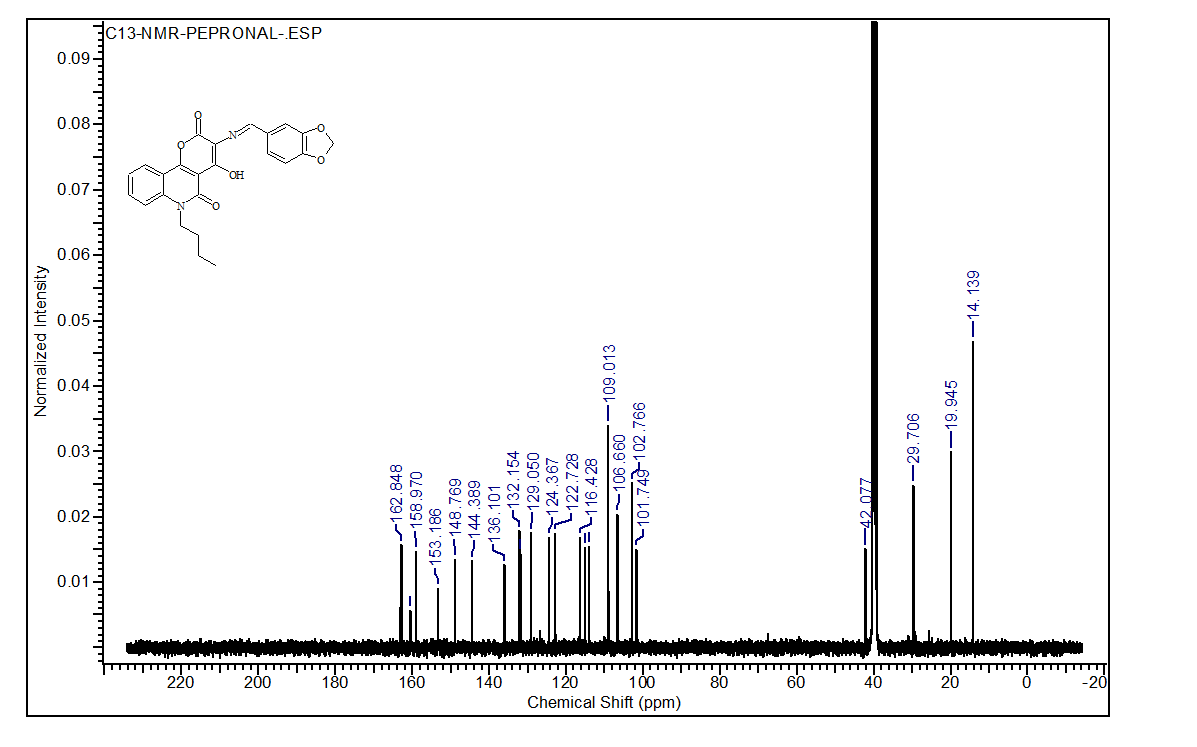
**

**^13^C-NMR** **Spectrum of compound no. 2g**

**
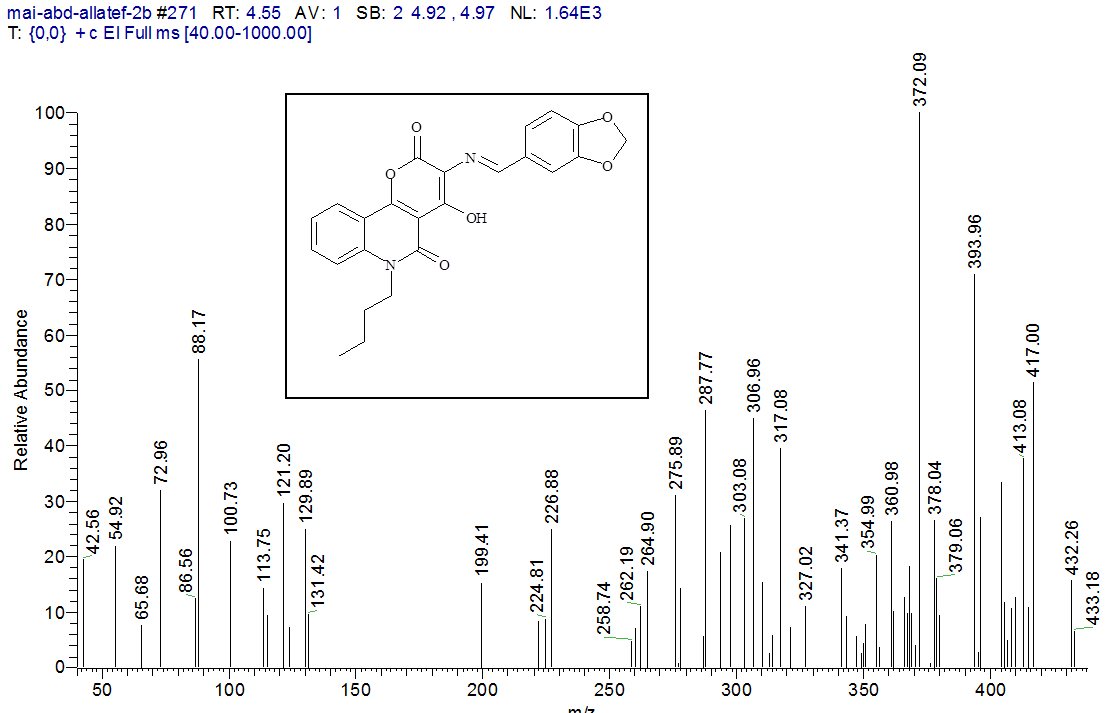
**
